# Lactic Acid Bacteria-Derived γ-Aminobutyric Acid: From Targeted Screening and Biosynthesis to Functional Food Applications and Health Benefits

**DOI:** 10.3390/foods15152642

**Published:** 2026-07-28

**Authors:** Yuqian Zhang, Xue Zhou, Dan Zheng, Xuezhi Yuan, Shuyun Xu, Jiangyu Zhu, Weiwei Cheng

**Affiliations:** 1 School of Chemistry and Bioengineering, Nanjing Normal University Taizhou College, Taizhou 225300, China; 20121008@nnutc.edu.cn (Y.Z.); 20081087@nnutc.edu.cn (X.Z.); 20091039@nnutc.edu.cn (D.Z.); 20051037@nnutc.edu.cn (X.Y.); 2 School of Food Science and Engineering, Yangzhou University, Yangzhou 225127, China; mx120251361@stu.yzu.edu.cn (S.X.); 008051@yzu.edu.cn (J.Z.)

**Keywords:** γ-aminobutyric acid, lactic acid bacteria, glutamate decarboxylase, high-throughput screening, metabolic engineering, functional foods

## Abstract

γ-Aminobutyric acid (GABA) is a non-proteinogenic amino acid that acts as a major signaling molecule across the nervous, cardiovascular, and immune systems. While GABA has historically been produced via chemical synthesis or plant extraction, microbial fermentation using lactic acid bacteria (LAB) provides a safe, sustainable, and food-grade alternative. This review details the recent progress of LAB-derived GABA, covering the workflow from strain selection to functional food applications. We discuss how modern screening methods combine high-throughput phenotypic testing with genomic mining of the *gad* operon to efficiently identify high-yielding strains. The biochemical mechanisms of the GABA shunt are also explained, alongside recent CRISPR-based metabolic engineering efforts designed to bypass natural yield limits. Furthermore, we address practical industrial challenges—such as the poor proteolytic ability of key producers like *Levilactobacillus brevis*—and evaluate viable solutions, including symbiotic co-cultures and optimized downstream purification steps. The review then summarizes the specific health benefits of dietary LAB-derived GABA, focusing on its ability to relieve anxiety via the microbiota-gut–brain axis, control blood pressure, and regulate immunity. Finally, we analyze the current regulatory and sensory hurdles, highlighting how integrating multi-omics data can help establish LAB-derived GABA as a reliable ingredient for functional foods and personalized nutrition.

## 1. Introduction

γ-Aminobutyric acid (GABA) is the primary inhibitory neurotransmitter in the mammalian central nervous system. It binds to ionotropic GABA_A_ and metabotropic GABA_B_ receptors, altering neuronal ion permeability to produce sedative, anxiolytic, and anticonvulsant effects [1]. Beyond the brain, increasing clinical and epidemiological data indicate that GABA and GABA-enriched dietary products offer various peripheral health benefits, such as lowering blood pressure, regulating gastrointestinal motility, and modulating immune responses [2,3]. Because of its therapeutic potential, the food and pharmaceutical industries have shown growing interest in GABA, creating a need for more efficient and scalable production methods.

The acquisition of GABA historically relied on direct extraction from natural plant and animal tissues, where its basal concentrations are intrinsically low and insufficient for commercial exploitation. To address this issue, contemporary production strategies have diverged into chemical synthesis, plant enrichment, and microbial fermentation. Chemical synthesis relies on severe reaction conditions, such as the high-temperature substitution of γ-chloroprene cyanide with potassium phthalimide, followed by aggressive acidic hydrolysis [4]. While capable of producing GABA, this approach involves toxic precursors and generates hazardous chemical residues, strictly precluding its application in the food and nutraceutical sectors where purity and safety are paramount. Conversely, plant enrichment leverages abiotic stressors—such as hypoxia, cold shock, and low-frequency ultrasound—to release the autoinhibitory domain of endogenous glutamate decarboxylase (GAD), thereby triggering intracellular GABA accumulation [5]. Despite its environmental benignity, plant-based enrichment is frequently hindered by complex downstream extraction processes, matrix interference, and overall low product yield [4,5].

In contrast, microbial biosynthesis via the GABA shunt pathway provides a robust, sustainable, and highly efficient alternative. The core mechanism involves the irreversible α-decarboxylation of L-glutamate, catalyzed by the cytosolic enzyme GAD with pyridoxal 5′-phosphate (PLP) serving as an obligatory cofactor [6]. Among the diverse microbial candidates capable of this conversion, lactic acid bacteria (LAB) have emerged as the most promising biocatalysts. LAB possess a “generally recognized as safe” (GRAS) status and are inherently food-grade, extensively utilized worldwide as starter cultures and probiotics [7,8]. Exploiting specific LAB strains—such as *Levilactobacillus brevis*, *Lactiplantibacillus plantarum*, and *Lactococcus lactis*—for GABA production bypasses the safety concerns associated with chemical synthesis while allowing the direct formulation of functional fermented dairy, cereal, and beverage products without the absolute necessity for exhaustive downstream purification [8]. Furthermore, the distinctive metabolic pathways of LAB, including the *gad* operon responsible for coupling glutamate decarboxylation with proton consumption, endow these strains with a unique mechanism to combat extracellular acid stress while simultaneously accumulating high titers of extracellular GABA [9].

Despite the evident advantages of LAB-derived GABA, translating this potential into standardized industrial applications encounters multifaceted challenges. The vast taxonomic diversity within LAB translates to significant inter- and intra-species variations in GAD enzymatic activity, proteolytic capacity, and environmental adaptability [10]. Consequently, isolating wild-type LAB strains with naturally superior GABA-producing phenotypes demands the transition from labor-intensive traditional cultivation to high-throughput and genome-driven targeted screening methodologies. Furthermore, unlocking the full biosynthetic potential of these strains necessitates precise metabolic engineering, process optimization, and comprehensive evaluation of their post-ingestion functional efficacies.

This review aims to summarize recent progress in the research and application of LAB-derived GABA. We outline current targeted screening techniques, molecular mechanisms of GABA biosynthesis, and recent metabolic engineering strategies. In addition, we review the optimization of fermentation and purification processes, summarize the health benefits of LAB-derived GABA, and discuss the remaining challenges and future opportunities for its application in the food industry.

## 2. Targeted Screening of High GABA-Producing Lactic Acid Bacteria

The ubiquitous but minor distribution of GABA-producing microorganisms in natural ecosystems necessitates robust, highly sensitive screening methodologies to isolate strains with superior biosynthetic capacities. The inherent complexity of microbial colony morphology and the vast physiological diversity within lactic acid bacteria populations render traditional random isolation approaches inefficient and labor-intensive. Consequently, contemporary screening paradigms have shifted from conventional analytical techniques toward a systematic integration of high-throughput phenotypic assays and bioinformatics-driven genotypic evaluations.

### 2.1. High-Throughput Phenotypic Screening (HTS) Methodologies

Traditional analytical tools for GABA quantification, such as thin-layer chromatography (TLC) and high-performance liquid chromatography (HPLC), are generally time-consuming and require complex sample derivatization [11,12]. To speed up the initial strain isolation process, researchers have developed several high-throughput screening assays. For example, the GABase microtiter plate assay uses a dual-enzyme system (GABA-T and SSADH). This system facilitates the conversion of GABA to succinic semialdehyde (SSA), coupled with the reduction in NADP^+^ to NADPH, permitting the spectrophotometric quantification of GABA without prior derivatization [13]. However, from a critical limitation of this enzymatic assay is the high potential for background optical interference at 340 nm. Complex food and fermentation matrices inherently contain soluble proteins, polyphenols, and carotenoids that absorb strongly in the UV-to-near-UV spectrum, frequently leading to overestimation of GABA yields unless rigorous blanking or sample pre-treatment is employed. Similarly, the sensitivity-intensified ninhydrin-based chromogenic system (SINICS) has been optimized for rapid, cost-effective, and high-throughput relative quantification of GABA directly from fermentation broths [14]. Nevertheless, a critical analytical challenge is that residual L-glutamate (the primary substrate) strongly interferes with the ninhydrin-mediated color formation at 570 nm. Therefore, relying solely on direct chromogenic readings is often inaccurate; reliable quantification strictly requires adequate sample separation—such as thin -layer chromatography (TLC) or solid-phase extraction—prior to spectrophotometric evaluation. Additionally, exploiting the stoichiometric generation of carbon dioxide and consumption of intracellular protons during the glutamate decarboxylation process, indirect screening models have been conceptualized. Assays utilizing pH indicators (e.g., bromocresol purple) or measuring gas-release volumes within micro-bioreactors provide immediate visual or physical parameters correlating to GAD enzymatic activity, significantly accelerating the isolation of highly active candidates from complex environmental matrices like fermented foods and feces [11,15]. Integrating these optical and physical high-throughput strategies with advanced robotic colony-picking workstations and droplet-based microfluidic fluorescence-activated cell sorting (FACS) effectively eliminates subjective bias and escalates the scale of phenotypic evaluation to millions of variants per cycle [16].

### 2.2. Genotypic Screening via Comparative Genomics

While phenotypic screening verifies functional outputs, it is inherently restricted by the reliance on specific cultivation conditions that might suppress the expression of biosynthetic gene clusters. To address this limitation, genotypic screening utilizing molecular probes and comparative genomics provides a cultivation-independent, highly specific avenue for microbial bioprospecting [16,17].

The core genetic determinant for GABA biosynthesis in LAB is the *gad* operon, fundamentally encoding the glutamate decarboxylase enzyme(s) (*gadA* and/or *gadB*) and the strictly coupled glutamate/GABA antiporter (*gadC*) [18]. By employing colony polymerase chain reaction (PCR) with degenerate primers designed against highly conserved active-site domains (e.g., the PLP-binding domain) of LAB GADs, researchers can rapidly verify the intrinsic genetic capability of uncultured or newly isolated strains [19]. With the exponential expansion of whole-genome sequencing (WGS) databases, sophisticated bioinformatics tools facilitate the in silico prediction of probiotic properties. Large-scale genomic surveys indicate that the complete structural integrity of the *gad* operon (*gadCBA*) serves as a reliable molecular marker for distinguishing elite GABA producers. For instance, comparative genomic analyses have demonstrated that strains lacking the *gad* operon or possessing truncated variants intrinsically fail to accumulate significant extracellular GABA, irrespective of substrate supplementation [18,20]. Thus, leveraging genomic datasets to pinpoint sequence homology and operon completeness fundamentally streamlines the identification of optimal cellular factories.

### 2.3. Species Diversity and Ecological Distribution

Ecological surveillance reveals that elite GABA-producing LAB are predominantly localized within acidic environments characterized by high osmotic stress and abundant proteinaceous precursors, such as traditional fermented vegetables (e.g., kimchi, paocai), artisanal cheeses, and human or animal intestinal tracts [11,21]. The stringent selective pressure of these microenvironments inherently drives the retention and high expression of the *gad* operon, which operates as a critical acid-resistance mechanism by eliminating intracellular protons [11].

Taxonomically, profound interspecies variations dictate the capacity for GABA synthesis. As summarized in Table 1, the most potent GABA producers are frequently identified within the species *Levilactobacillus brevis* (basonym: *Lactobacillus brevis*), *Lactiplantibacillus plantarum* (basonym:* Lactobacillus plantarum*), *Lactococcus lactis*, and *Streptococcus thermophilus* [11,21,22]. *Lv. brevis* exhibits exceptional biosynthetic superiority; genomic evaluations establish that nearly 100% of completely sequenced *Lv. brevis* isolates harbor an intact *gad* operon accompanied by two functionally distinct GAD-encoding genes, making it an unparalleled candidate for industrial exploitation [11,18]. Conversely, dairy-associated isolates like *S. thermophilus* and *Lc. lactis*, though generally yielding lower absolute GABA concentrations compared to *Lv. brevis*, offer profound advantages in milk fermentation systems owing to their robust proteolytic nature, which naturally supplies the glutamate precursor essential for in situ GABA generation [11,23].

## 3. Biosynthetic Mechanisms and Genetic Engineering of GABA in LAB

### 3.1. Deciphering the GABA Shunt and the Glutamate Decarboxylase System

In LAB, the accumulation of extracellular GABA is predominantly driven by the GABA shunt, a highly conserved metabolic bypass linking amino acid metabolism with intracellular acid resistance. Mechanistically, this pathway relies on the synergistic function of the GAD (EC 4.1.1.15) enzyme and the membrane-bound glutamate/GadC, both of which are transcriptionally regulated within the *gad* operon [21].

The biosynthetic cascade is initiated by the active transport of exogenous L-glutamate into the bacterial cytoplasm via the GadC antiporter. Subsequently, the cytosolic GAD enzyme catalyzes the irreversible α-decarboxylation of L-glutamate to yield GABA. This specific decarboxylation event strictly requires PLP as a co-factor and consumes one intracellular proton (H^+^) while releasing one molecule of carbon dioxide (CO_2_) [28]. The newly synthesized GABA is subsequently extruded back into the extracellular matrix via GadC in exchange for another glutamate molecule. From a physiological perspective, this proton-consuming mechanism serves as a critical fitness determinant, effectively mitigating cytosolic acidification and enabling LAB to maintain pH homeostasis under extreme fermentative conditions [28,29]. Consequently, the transcriptional activation of the *gad* operon is fundamentally tightly coupled with the environmental pH, triggering explosive GABA synthesis primarily when the extracellular matrix reaches critical acidity.

### 3.2. Biochemical Kinetics and Phylogenetic Diversity of LAB Glutamate Decarboxylases

The intrinsic capacity of distinct LAB strains to synthesize GABA is dictated by the structural configuration and catalytic kinetics of their specific GADs. Phylogenetic analyses based on the amino acid sequences of translated GAD proteins reveal highly conserved evolutionary lineages at the species level, specifically clustered within *Levilactobacillus brevis*, *Lactiplantibacillus plantarum*, and *Lactococcus lactis* [11]. Notably, genomic mining indicates that *Lv. brevis* harbors a unique genetic architecture characterized by the presence of two functionally distinct *gadA* and *gadB* alongside an intact *gad* operon, partially explaining its superior biosynthetic capabilities compared to other LAB [11].

Catalytic profiling of heterologously expressed and purified GADs from various LAB elucidates significant variations in enzyme kinetics (Table 2). The optimal catalytic activity for the vast majority of LAB-derived GADs is strictly confined to an acidic range (pH 4.0 to 5.2). This acidic optimum creates an operational paradox during industrial fermentation: while low pH maximizes GAD specific activity, it simultaneously impedes cellular proliferation and diminishes overall bacterial viability [6,11]. Furthermore, the optimal temperatures for purified GADs frequently exceed 45 °C (e.g., 50 °C for *Lacticaseibacillus paracasei* NFRI7415 and 55 °C for *Streptococcus thermophilus* Y2), which deviates substantially from the standard mesophilic growth temperatures (30–37 °C) of these strains. The variations in the Michaelis constant (*K_m_*)—ranging from highly affine enzymes (*K_m_* = 0.5 mM in *Lc. lactis* 01-7) to those requiring high substrate concentrations (*K_m_* = 22.8 mM in *Lb. plantarum* ATCC 14917)—further underscore the necessity of customizing the fermentation matrix and precursor feeding strategies to match the specific kinetic profile of the engineered or selected strain [30,31].

### 3.3. Rewiring GABA Biosynthesis via Advanced Genetic Engineering and CRISPR Technologies

**Figure 1:**
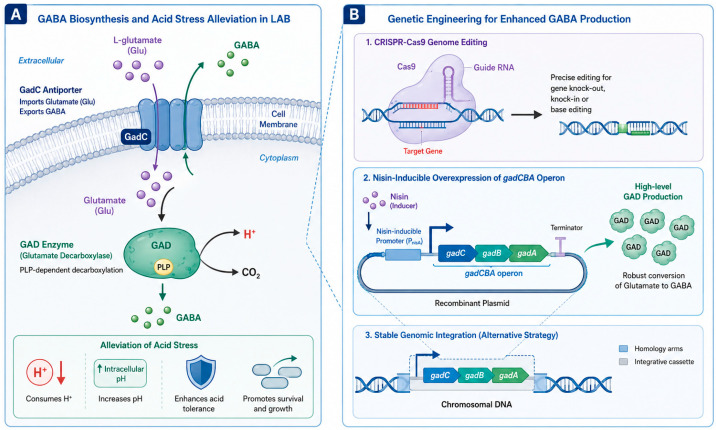
Biosynthetic mechanisms and genetic engineering of GABA in lactic acid bacteria. (**A**) The intracellular GABA shunt pathway mediated by the glutamate/GadC and GAD, coupling glutamate conversion with proton consumption to mitigate acid stress. (**B**) Targeted metabolic rewiring using CRISPR-Cas genomic editing systems to seamlessly overexpress the *gad* operon and optimize carbon flux for enhanced GABA accumulation.

While optimizing fermentation parameters can improve baseline GABA yields, metabolic engineering is often necessary to overcome natural metabolic bottlenecks. Traditional genetic modifications in LAB mostly relied on vector-based homologous recombination (HDR), using double-crossover events for gene knockouts or replacements (Figure 1). For example, knocking out the transcriptional regulator *gadR* in *Lv. brevis* completely stopped GABA production, confirming its role as a positive regulator of the *gadCB* operon [38]. Similarly, overexpressing the endogenous *gadB* and *gadC* genes under a nisin-inducible promoter significantly increased GABA titers compared to using wild-type promoters [39].

However, traditional HDR often suffers from low recombination efficiencies and the unwanted retention of antibiotic resistance markers. To address these issues, CRISPR-Cas systems have been adapted for LAB genome editing. Advanced tools, such as the RecE/T-assisted CRISPR-Cas9 platform, have been successfully used in historically difficult-to-edit strains like *Lv. brevis* and *Lb. plantarum*, achieving gene deletion and replacement with near 100% efficiency [40]. Furthermore, researchers are using CRISPR interference (CRISPRi) with a catalytically inactive Cas9 (dCas9) to physically block RNA polymerase. By repressing competing pathways (such as lactate dehydrogenase or polyamine degradation) and simultaneously overexpressing the *gad* operon, CRISPRi effectively redirects carbon and nitrogen fluxes toward GABA biosynthesis [41]. These genetic tools allow researchers to construct high-yield, food-grade LAB cell factories without leaving foreign genetic sequences in the final strain (Figure 1).

## 4. Optimization of Fermentation, Isolation, and Purification Processes

Unlocking the maximum biosynthetic potential of LAB strictly necessitates the systematic optimization of both upstream fermentative parameters and downstream processing technologies. The transition from laboratory-scale confirmation to industrial-scale application relies on balancing physiological cell viability with maximal GAD catalytic efficiency, followed by rigorous purification to meet functional food and pharmaceutical standards.

### 4.1. Modulation of Critical Fermentation Parameters

The accumulation of GABA in microbial systems is not a static trait but rather a highly dynamic metabolic response governed by environmental conditions, particularly pH, temperature, and nutrient matrix compositions [21]. Because the decarboxylation of L-glutamate consumes intracellular protons, the GAD system functions optimally under acidic stress. While purified LAB GAD enzymes typically display maximum activity at a pH range of 4.0 to 5.0, maintaining the entire fermentation matrix at such extreme acidity drastically compromises cellular proliferation [11]. Consequently, dynamic pH control strategies, such as two-stage fermentation—where initial neutral conditions promote rapid biomass accumulation, followed by a controlled shift to acidic conditions to trigger explosive GABA synthesis—have proven highly effective [21].

Temperature equally dictates the metabolic flux, with optimal ranges demonstrating significant inter-species variability. While thermophilic strains like *Streptococcus thermophilus* achieve optimal synthesis near 40 °C, mesophilic producers such as *Levilactobacillus brevis* require strict temperature regulation (around 30–34 °C) to prevent the thermal denaturation of GAD [21]. The composition of the nutrient matrix, specifically the availability of the strictly required cofactor PLP and the substrate monosodium glutamate (MSG), acts as a primary limiting factor. Exceeding the osmotic tolerance threshold of the specific strain via excessive MSG supplementation inevitably inhibits cellular growth. Furthermore, carbon source substitution critically alters regulatory networks; replacing glucose with xylose has been documented to activate the *xyl* operon in *Lv. brevis*, fundamentally enhancing growth rates and resulting in a remarkable amplification of final GABA titers [21,25]. High-hydrostatic pressure treatments (e.g., 300 MPa) during cultivation have also been explored as a biophysical intervention to transiently alter membrane permeability, thereby enhancing substrate influx and product extrusion without causing permanent cellular damage [21].

### 4.2. Compensatory Strategies for Non-Proteolytic LAB

Although specific strains, particularly *Lv. brevis*, produce high amounts of GABA, they are difficult to use as primary starter cultures in dairy products because they lack proteolytic activity. Genomic analyses confirm that sequenced *Lv. brevis* strains do not have genes encoding cell envelope proteinases (CEPs, such as PrtP) [11]. This genetic absence prevents the bacteria from breaking down milk casein into usable oligopeptides and free amino acids, resulting in restricted growth and failed fermentation in unsupplemented milk matrices [11,42].

**Figure 2:**
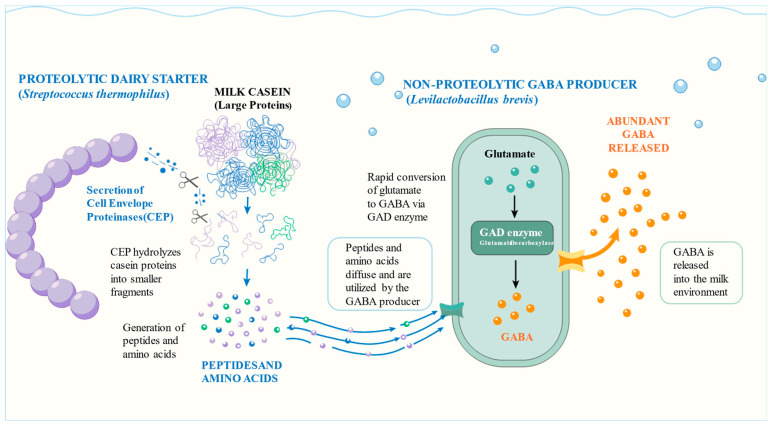
Symbiotic co-culture strategy for enhanced GABA production in dairy fermentation. Synergistic interactions between proteolytic dairy starters and non-proteolytic high-GABA producers (e.g., *Levilactobacillus brevis*). The enzymatic hydrolysis of milk caseins by CEPs from the primary starter liberates essential oligopeptides, effectively cross-feeding the GABA-producing adjunct and sustaining its metabolic flux within the milk matrix.

To solve this problem and produce naturally GABA-enriched fermented dairy products, researchers have developed two main approaches. The first approach is exogenous supplementation, which involves adding pre-hydrolyzed nitrogen sources—such as concentrated whey permeate, yeast extract, or germinated soybean extract—to the milk base. This provides the necessary small peptides (typically 4–20 amino acids) required to support *Lv. brevis* growth [11]. The second approach relies on symbiotic co-culture. This method pairs the non-proteolytic, GABA-producing adjunct (*Lv. brevis*) with traditional proteolytic dairy starters (e.g., *S. thermophilus* or *Lactococcus lactis*). In this mixed-culture system, the proteolytic starter breaks down milk caseins, constantly releasing essential peptides that cross-feed the GABA producer. Studies on the interaction between *S. thermophilus* and *Lv. brevis* in skim milk have shown improved viability of the adjunct strain (Figure 2) and higher final GABA concentrations, creating a functional dairy product without needing synthetic additives [21,42].

### 4.3. Advanced Technologies for Quantification, Isolation, and Purification

Accurate quantification and high-purity extraction from complex fermentation broths constitute the final critical phases of GABA production. Owing to the absence of a natural chromophore or fluorophore in the GABA molecule, direct detection via standard ultraviolet (UV) spectroscopy is impossible. Chromatographic quantification via HPLC remains the gold standard, mandating pre-column derivatization using reagents such as o-phthalaldehyde (OPA), dansyl chloride, or phenyl isothiocyanate (PITC) to facilitate detection [15,21]. For rapid, high-throughput relative quantification, the enzyme-linked GABase microtiter plate assay—measuring the NADPH-dependent absorbance shift during the enzymatic conversion of GABA to succinic acid—provides a precise, derivatization-free alternative [13]. Concurrently, the SINICS optimizes traditional TLC principles into a scalable spectrophotometric assay suitable for rapid industrial monitoring [14].

The downstream purification of extracellular GABA from crude microbial broth mandates the sequential elimination of residual carbohydrates, proteins, pigments, and unreacted glutamate. Industrial-scale isolation generally commences with flocculation and ultrafiltration to precipitate macromolecular impurities and cellular debris. The clarified permeate is subsequently subjected to decolorization utilizing activated carbon or macroporous adsorption resins (e.g., DA-201CII) [21]. To achieve pharmaceutical or functional food-grade purity, the decolorized solution undergoes ion exchange chromatography utilizing specific cation exchange resins (e.g., Amberlite 200C or QY-HG01) designed to separate GABA from structurally similar amino acids based on distinct isoelectric points [21,43]. The final concentration and refinement are typically accomplished via repeated ethanol precipitation and controlled crystallization, yielding colorless, stable GABA crystals with documented purities exceeding 98–99%, perfectly satisfying stringent commercial standards [21]. For post-purification structural validation, researchers frequently employ Fourier-transform infrared (FTIR) spectroscopy; however, FTIR claims should be strictly limited to identifying qualitative changes in specific vibrational modes (e.g., amine and carboxyl group stretches). To rigorously verify molecular weight and assess the absolute purity of the extract—particularly to rule out the co-precipitation of macro-molecular contaminants such as microbial exopolysaccharides (EPS)—advanced analytical techniques like size-exclusion chromatography coupled with multi-angle laser light scattering (SEC-MALLS) or gel permeation chromatography (GPC) are strictly required.

## 5. Physiological Functions and Health Benefits of LAB-Derived GABA

While historically defined exclusively as the principal inhibitory neurotransmitter within the mammalian central nervous system (CNS), GABA is now recognized as a highly pleiotropic signaling molecule orchestrating a vast array of peripheral physiological networks. The oral administration of purified GABA or the consumption of GABA-enriched functional foods—particularly those bio-transformed by LAB—has been explicitly linked to profound multi-target health benefits, spanning neuroprotection, hemodynamic regulation, and immunometabolic homeostasis.

### 5.1. Neuromodulation and the Microbiota-Gut–Brain Axis

In the CNS, GABA reduces neuronal excitability by binding to ionotropic GABA_A_ and metabotropic GABA_B_ receptors. This binding promotes physical relaxation, reduces anxiety, and improves sleep quality. Clinical studies indicate that dietary supplementation with microbial-derived GABA can increase α-wave activity and decrease β-wave activity in the human brain. This helps relieve psychological stress and insomnia symptoms without the adverse side effects typically associated with synthetic sleep medications like benzodiazepines [21,44].

Because dietary GABA exhibits highly limited permeability across the blood–brain barrier (BBB), its physiological anxiolytic and neurobehavioral effects cannot be simply attributed to direct central nervous system (CNS) infiltration. Instead, these effects are fundamentally driven by the microbiota-gut–brain axis. Specifically, LAB-derived GABA acts locally by activating enteric GABA receptors GABA_A_ and GABA_B_ distributed extensively within the gastrointestinal mucosa and the enteric nervous system (ENS) [45]. This enteric receptor activation subsequently modulates afferent vagal nerve signaling ascending to the brain. It is this critical transduction of localized enteric signaling via the vagus nerve—rather than the direct physical translocation of the GABA molecule—that dampens abnormal neuro-excitability, ultimately bridging luminal microbial metabolism with the alleviation of host emotional stress and anxiety [46,47].

### 5.2. Cardiovascular Protection and Hemodynamic Regulation

The peripheral GABAergic system plays a vital role in cardiovascular protection, specifically operating as a potent biological antihypertensive mechanism. The hemodynamic regulatory effects of GABA are mediated divergently across distinct receptor subtypes located within cerebrovascular and peripheral tissues. The activation of endothelial GABA_A_ receptors directly promotes vasodilation, thereby physically reducing resistance to vascular blood flow [21]. Conversely, the stimulation of presynaptic GABA_B_ receptors effectively inhibits the excitation of sympathetic nerve endings, suppressing the excessive release of catecholamines that drive hypertensive states [21].

Extensive in vivo evaluations employing spontaneously hypertensive rat (SHR) models confirm that the ingestion of GABA mitigates blood pressure fluctuations, regulates heart rate, and improves arterial baroreceptor reflex functionality [21]. Translating these physiological mechanisms into clinical efficacy, pioneering double-blind human trials have validated the therapeutic potential of functional dairy products. Daily intake of LAB-fermented milk containing physiological doses of GABA (e.g., 10–12 mg/100 mL) induced a significant and sustained reduction in systolic and diastolic blood pressure among mildly hypertensive patients within several weeks of intervention, underscoring the viability of LAB-derived GABA as a non-pharmacological dietary therapy for cardiovascular risk management [3].

### 5.3. Gastrointestinal Homeostasis and Immunometabolic Regulation

The enteric nervous system (ENS), an intricate network of autonomous neurons within the intestinal wall, is heavily regulated by local GABAergic signaling. Mucosal endocrine-like cells and myenteric plexus neurons utilize GABA to modulate both cholinergic and non-adrenergic non-cholinergic (NANC) neurotransmission [48]. By activating enteric GABA_A_ and GABA_B_ receptors, luminal GABA meticulously controls spontaneous gastrointestinal relaxations and contractions, optimizing gastric emptying and intestinal peristalsis. This targeted neuromodulation positions GABA as a promising therapeutic target for treating functional gastrointestinal motor disorders, such as gastroparesis and acute colonic pseudo-obstruction [21,48].

**Figure 3:**
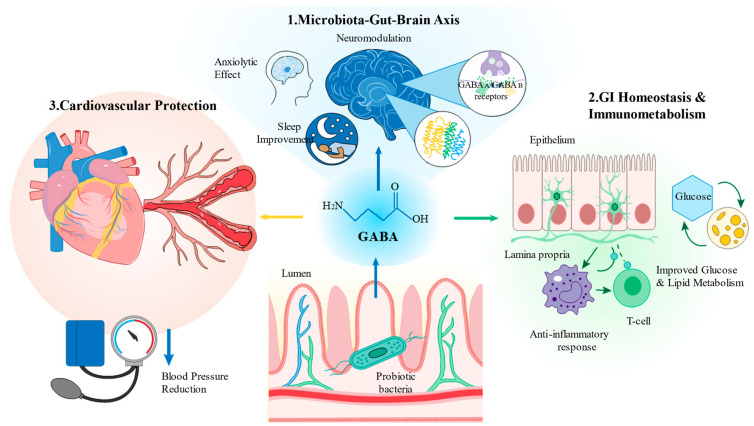
Multi-target physiological functions and systemic health benefits of LAB-derived GABA. Dietary intervention with microbial GABA or GABA-enriched functional foods exerts pleiotropic therapeutic effects. The key pathways include modulating the microbiota-gut–brain axis to alleviate neurological and psychiatric disorders, attenuating cardiovascular hypertension via endothelial vasodilation and sympathetic inhibition, and orchestrating gastrointestinal motility and immunometabolic homeostasis.

Furthermore, recent immunological paradigms reveal that peripheral immune cells—including macrophages, dendritic cells, and T lymphocytes—possess functional GABA receptor complexes [21,49]. The binding of GABA to these immune-associated receptors triggers distinct anti-inflammatory cascades. Experimental evidence establishes that GABAergic activation actively down-regulates the secretion of pro-inflammatory cytokines while simultaneously suppressing the excessive proliferation of autoreactive T cells [49]. Consequently, systemic or gut-derived GABA exerts protective immunomodulatory effects, actively ameliorating the pathophysiological progression of systemic autoimmune conditions, notably type 1 diabetes mellitus, rheumatoid arthritis, and severe mucosal inflammation [21,49]. Concurrently, specific GABA-enriched dietary interventions display profound metabolic benefits, actively maintaining blood glucose homeostasis, improving glycolipid metabolism, and reversing gut microbiota dysbiosis in type 2 diabetic models, representing a holistic approach to managing metabolic syndrome [21] (Figure 3).

## 6. Functional Food Applications, Challenges, and Future Perspectives

### 6.1. Industrial Applications in Functional Foods and Beyond

The translation of LAB-derived GABA from laboratory bioprocesses to commercial food matrices represents a highly dynamic sector within the global nutraceutical market. Recognizing its physiological safety and neuroactive potential, regulatory authorities globally have progressively endorsed GABA as a legitimate dietary component; notably, the Chinese Ministry of Health officially approved GABA as a new resource food in 2009, stipulating a maximum daily intake of 500 mg [21]. Beyond China, GABA has been extensively integrated into global regulatory frameworks. In Japan, it is one of the most frequently notified active ingredients approved under the Foods with Function Claims (FFC) and Foods for Specified Health Uses (FOSHU) systems, widely utilized in functional beverages and snacks for stress relief. In the United States, GABA is regulated as a dietary supplement under the Dietary Supplement Health and Education Act (DSHEA) and has achieved Generally Recognized as Safe (GRAS) status by the FDA for inclusion in diverse food matrices. Similarly, the European Union permits the use of GABA in food supplements, albeit subject to specific national guidelines and EFSA evaluations. This regulatory validation has catalyzed the incorporation of microbial GABA into a diverse array of functional products. Specifically, high GABA-producing LAB isolates are deployed as adjunct starter cultures to manufacture naturally enriched fermented dairy products, such as yogurt and artisanal cheeses, bypassing the need for exogenous synthetic chemical additions [11,50]. Beyond traditional dairy, LAB-mediated GABA enrichment has been successfully extended to plant-based matrices, yielding functional beverages, fermented soy products (e.g., tempeh), and specialty confections like dark chocolate, where the inclusion of GABA concurrently enhances moisture retention and mitigates textural degradation [11,21].

Extending beyond human nutrition, microbial-derived GABA exhibits profound applicability within the agricultural and livestock sectors. Exogenous GABA application in crop management effectively enhances abiotic stress tolerance, mitigating heavy metal accumulation and preserving postharvest quality [21]. Concurrently, dietary GABA supplementation in livestock husbandry provides a robust strategy to alleviate heat-induced oxidative stress and behavioral aberrations in intensively reared domestic animals, improving overall immunological resilience and physiological performance without relying on pharmaceutical interventions [21].

### 6.2. Technical, Sensory, and Regulatory Challenges

Despite the established commercial trajectory, the large-scale industrialization of LAB-derived GABA encounters distinct technical, sensory, and regulatory bottlenecks.

Sensory Impacts and Metabolic By-Products: A primary sensory challenge arises from the requisite substrate supplementation during fermentation. The addition of high concentrations of MSG to maximize GAD-mediated GABA conversion inevitably leaves unreacted residual glutamate in the final matrix [11]. Because glutamate operates as a potent umami flavoring agent, its excessive persistence severely disrupts the delicate organoleptic balance of fermented dairy and beverage products, shifting taste profiles toward savory notes that may be undesirable [11]. Furthermore, while high-purity GABA is generally odorless, high concentrations of accumulated GABA itself can introduce a mild bitter or astringent off-taste to the final product.

In the case of intensified GABA synthesis, metabolic by-products present another limitation. When LAB metabolic flux is heavily directed toward the GABA shunt under extreme acid stress, it alters intracellular redox and energy balances. This can drive the elevated production of volatile organic acids (e.g., excessive acetate or lactate) or flavor-active compounds (e.g., diacetyl) that further alter the product’s sensory profile. Additionally, if non-specific amino acid decarboxylases are natively co-expressed during stress conditions, trace amounts of biogenic amines (e.g., tyramine or histamine) could accumulate, raising food safety concerns that mandate rigorous quality control [10,11].

Regulation of GABA Synthesis in Food Systems: To address these limitations, regulating GABA synthesis in food systems is crucial, demanding distinct strategies for natural wild-type and genetically modified (GM) strains. For natural LAB, regulation relies heavily on bioprocess engineering. For example, two-stage pH-regulated fermentation prevents early growth inhibition while dynamically triggering localized GABA synthesis [21]. Step-wise MSG micro-feeding prevents osmotic shock, while symbiotic co-culturing with highly proteolytic strains (e.g., *Streptococcus thermophilus*) continuously supplies nitrogenous precursors, effectively minimizing unreacted MSG and bypassing the need for expensive nitrogen sources (like yeast extracts) that escalate production costs [11,42].

Conversely, for GM strains, GABA synthesis can be precisely regulated using food-grade inducible promoters (e.g., nisin-controlled systems) or pH-inducible endogenous promoters [39]. Tools like CRISPR interference (CRISPRi) can dynamically repress competing metabolic pathways and redirect flux strictly toward GABA accumulation during the late exponential phase, minimizing unwanted by-products without leaving foreign markers [40,41].

Regulatory and Safety Frameworks: Despite these technical advances, from a regulatory and safety perspective, the application of advanced metabolic engineering introduces profound ethical and legal complications. While CRISPR-Cas9 genome editing holds immense promise for constructing hyper-producing LAB cell factories, the potential for off-target sequence modifications unpredictably altering strain behavior remains a critical safety concern [51]. Furthermore, classical genetic modifications relying on plasmid-borne antibiotic resistance markers pose severe biohazard risks regarding horizontal gene transfer within the human gut microbiome post-consumption [11]. Consequently, the deployment of genetically modified LAB strains in commercial food formulations is strictly governed by rigorous safety evaluations, transparent regulatory oversight, and stringent ethical frameworks, frequently limiting their immediate application compared to wildly screened isolates [51].

### 6.3. Future Perspectives and Precision Nutrition

The future trajectory of LAB-derived GABA research is deeply intertwined with the evolution of multi-omics technologies and personalized medicine. Integrating whole-genome sequencing, transcriptomics, and metabolomics will unequivocally accelerate the discovery of cryptic biosynthetic gene clusters and elucidate the intricate molecular dialogues between specific GABA-producing probiotics and host intestinal epithelia [51]. By mapping the precise metabolic flux of these strains, bioprocess engineers can transition from empirical media formulation toward mathematically modeled, rationally designed fermentation matrices.

Clinically, the paradigm is shifting toward personalized probiotic interventions. Customizing LAB formulations based on an individual’s unique microbiome architecture and metabolic baseline offers unprecedented precision in alleviating specific neuro-metabolic disorders [51]. To substantiate these therapeutic claims and transition microbial GABA from a nutritional supplement to a rigorously validated biotherapeutic agent, future endeavors must prioritize large-scale, double-blind, placebo-controlled clinical trials spanning diverse demographic cohorts (Table 3). Concurrently, the development of green, solvent-free downstream purification technologies will be imperative to ensure that the ecological sustainability of microbial GABA production matches its physiological efficacy [21].

## 7. Conclusions

The production of GABA using LAB connects microbiological research, metabolic engineering, and functional food development. The natural ability of specific LAB strains to synthesize GABA, primarily driven by the proton-consuming *gad* operon, offers a food-grade alternative to chemical synthesis and plant extraction. Moving from traditional isolation methods to high-throughput screening and CRISPR-based gene editing has greatly expanded the production capacity of these strains. Both animal models and clinical studies confirm that dietary LAB-derived GABA provides diverse health benefits, including regulating the gut–brain axis, lowering blood pressure, and improving immune and metabolic health.

However, applying these findings to commercial products requires solving specific practical challenges. These include mitigating the negative sensory effects caused by residual glutamate in fermented foods and navigating the strict regulatory approval processes for genetically modified probiotics. By addressing these issues and utilizing multi-omics tools to better understand LAB metabolism, researchers can further integrate GABA-producing LAB into the development of next-generation functional foods and personalized nutrition.

## Figures and Tables

**Table 1:** Representative high GABA-producing lactic acid bacteria: Ecological origins, target yields, and integrated screening methodologies.

Species/Strain	Origin	GABA Yield	Screening & Analytical Methodology	Reference
*Levilactobacillus brevis* NCL912	Chinese paocai	35.66 g/L	Comparative genomics/HPLC	[20,24]
*Levilactobacillus brevis* NPS-QW-145	Korean kimchi	25.83 g/L	Gas release-based assay/RP-HPLC	[11,15]
*Levilactobacillus brevis* CE701	Fermented food	176.04 g/L	Genome mining/HPLC	[25]
*Lactiplantibacillus plantarum* NTU 102	Cabbage pickles	629 mg/L	Response surface screening/HPLC	[21,26]
*Lactobacillus sakei* B2-16	Rice bran extract	68.06 g/L	TLC/Amino acid analyzer	[21,27]
*Lactococcus lactis* B	Korean kimchi	6.41 g/L	Microtiter plate assay/HPLC	[22]
*Streptococcus thermophilus* Y2	Dairy samples	7.98 mg/L	pH indicator-based screening/HPLC	[11,23]

**Table 2:** Biochemical properties and kinetic parameters of heterologously expressed and purified GADs from representative lactic acid bacteria.

Strain	GAD Length (aa)	Optimal Conditions	Enzyme Kinetics (*K_m_*)	Reference
*Levilactobacillus brevis* IFO 12005	480	pH 4.2, 30 °C	9.3 mM	[32]
*Lactococcus lactis* 01-7	466	pH 4.7, 30 °C	0.5 mM	[33]
*Lacticaseibacillus paracasei* NFRI7415	481	pH 5.0, 50 °C	5.0 mM	[34]
*Streptococcus thermophilus* Y2	459	pH 4.0, 55 °C	2.3 mM	[35]
*Levilactobacillus brevis* CGMCC 1306	479	pH 4.8, 48 °C	10.26 mM	[36]
*Levilactobacillus brevis* 877G	468	pH 5.2, 45 °C	3.6 mM	[37]

**Table 3:** Summary of physiological functions and therapeutic outcomes of LAB-derived GABA or GABA-enriched functional foods in animal models and clinical trials.

Subject/Model	Intervention/Formulation	Dose & Duration	Physiological Function & Main Findings	Reference
Mildly hypertensive patients (Human trial)	GABA-rich fermented milk (by *Lactococcus lactis*)	100 mL/day (containing 10–12 mg GABA) for 12 weeks	Significant and sustained reduction in systolic and diastolic blood pressure.	[3]
Healthy adult males (Human trial)	GABA-enriched chocolate	10 g chocolate per dose (containing exactly 28 mg GABA)	Rapid reduction in psychological stress and mental fatigue during task performance.	[52]
Patients with insomnia symptoms (Human trial)	Fermented rice germ extract containing GABA	300 mg/day for 4 weeks	Decreased sleep latency and improved overall sleep efficacy without adverse effects.	[53]
Spontaneously hypertensive rats (Animal model)	GABA-enriched tempeh-like fermented soybean	0.3 mg GABA per rat per day	Retarded progressive elevation of blood pressure in hypertensive models.	[54]
Type 2 diabetic mice (Animal model)	GABA-rich germinated adzuki beans	Dietary inclusion at 0.1 g GABA per kg of diet for 6 weeks	Decreased fasting blood glucose, improved glycolipid metabolism.	[55]
Visceral hypersensitivity rats (Animal model)	*Lactococcus lactis* NCDO2118 (GABA producer)	1 × 10^9^ CFU/day for 15 days	Significant attenuation of stress-induced visceral pain and abdominal discomfort.	[45]
Anxiety-model mice (Animal model)	*Lactiplantibacillus plantarum* LZU-J-TSL6	4 × 10^8^ CFU/kg/day for 7 days (post-modeling)	Anxiolytic and antidepressant-like behavioral responses; improved stress resilience.	[47]

## Data Availability

No new data were created or analyzed in this study. Data sharing is not applicable.
